# Development of a Chinese werewolf deception database

**DOI:** 10.3389/fpsyg.2022.1047427

**Published:** 2023-01-09

**Authors:** Chaocao Yang, Xuqun You, Xudong Xie, Yuanyuan Duan, Buxue Wang, Yuxi Zhou, Hong Feng, Wenjing Wang, Ling Fan, Genying Huang, Xunbing Shen

**Affiliations:** ^1^Key Laboratory of Psychology of TCM and Brain Science, Jiangxi Administration of Traditional Chinese Medicine, Jiangxi University of Chinese Medicine, Nanchang, China; ^2^School of Psychology, Shaanxi Normal University, Xi’an, China; ^3^Shaanxi Provincial Key Laboratory of Behavior and Cognitive Neuroscience, Shaanxi Normal University, Xi’an, China

**Keywords:** deception database, facial expression, video, ecological validity, cross-culture, individual difference, emotion

## Abstract

Although it is important to accurately detect deception, limited research in this area has been undertaken involving Asian people. We aim to address this gap by undertaking research regarding the identification of deception in Asians in realistic environments. In this study, we develop a Chinese Werewolf Deception Database (C2W2D), which consists of 168 video clips (84 deception videos and 84 honest videos). A total of 1,738,760 frames of facial data are recorded. Fifty-eight healthy undergraduates (24 men and 34 women) and 26 drug addicts (26 men) participated in a werewolf game. The development of C2W2D is accomplished based on a “werewolf” deception game paradigm in which the participants spontaneously tell the truth or a lie. Two synced high-speed cameras are used to capture the game process. To explore the differences between lying and truth-telling in the database, descriptive statistics (e.g., duration and quantity) and hypothesis tests are conducted using action units (AUs) of facial expressions (e.g., *t*-test). The C2W2D contributes to a relatively sizable number of deceptive and honest samples with high ecological validity. These samples can be used to study the individual differences and the underlying mechanisms of lies and truth-telling between drug addicts and healthy people.

## 1. Introduction

Deception is an intentional attempt to mislead others (DePaulo et al., 2003). The deceptive behaviors occur in the form of intended lies, fabrications, omissions, and misrepresentations. People lie to escape from a situation that seems unfavorable to them (Hartman et al., 2022). Some lies are detrimental and threaten social stability and national security (Wu et al., 2018). Misreading deception or wrongful assertions of deceit can lead to exclusion, loss of resources, embarrassment, or termination of existing relationships (Carton et al., 1999; Belot et al., 2010). Although the need to accurately detect deception is essential, the observers only perform slightly better as compared with a random assertion, when deciding whether a person is lying, and the average accuracy is only 54% (Vrij et al., 2000; Bond and DePaulo, 2006).

The poor accuracy, to some degree, stems from the lack of reliable deception cues that are consistent across various situations (Levine, 2014). The studies have examined potential deceptive cues for detecting deception, including physiological cues [e.g., Polygraph (Iacono and Ben-Shakhar, 2019)], thermal imaging (Harper et al., 2019), electroencephalography (Kohan et al., 2020), functional magnetic resonance imaging (Yu et al., 2019), cognitive cues [e.g., cognitive load (Vrij et al., 2012)], memory confabulation (Dianiska et al., 2019)], and the behavioral cues (DePaulo et al., 2003; Hauch et al., 2015). However, the physiological and cognitive cues cannot be used in a contactless style, resulting in the inability to spot liars in public areas, such as airports or subway stations.

The research demonstrates that some behavioral cues, especially facial cues, may differentiate between lying and being truthful (Vrij et al., 2000; Shen et al., 2021; Wang et al., 2021). Darwin noted that some facial muscles are difficult to control especially when one feels strong emotions and they can show one’s authentic feelings (Ekman, 2003). Porter and ten Brinke (2008) also reported that one inconsistent expression is leaked transiently at least once, while an unfelt emotional facial expression is fake or liars neutralize an emotion. In short, facial cues can be a potentially effective indicator of deception.

Recently, researchers have shown increasing interest in automatically detecting deceptive behavior cues by applying machine learning and computer vision techniques (Meservy et al., 2005, 2008; Jensen et al., 2010; Michael et al., 2010; Wright et al., 2012; Borza et al., 2018; Chen et al., 2018; Shen et al., 2021). Studies have shown that some facial features can be important cues for detecting deception, including blink rate (Borza et al., 2018), facial movements (Chen et al., 2018), and facial expressions (Shen et al., 2021). These facial features are employed in distinguishing lies from the truth with relatively high accuracy. Nevertheless, most of the research has been conducted in the laboratory. A few studies in which real-life deception datasets are used have a small number of samples [real-life trial, refer to Shen et al. (2021)] that negatively affect the prediction rule in machine learning. Therefore, a more extensive dataset with high ecological validity is urgently needed for automatic deception detection.

In terms of ecological validity, the focus has primarily been on producing deception. Most deception research is conducted in laboratories that differ from natural conditions (Orne and Holland, 1968; Levine, 2018; Kihlstrom, 2021). The experimental instructions drastically limit the pragmatic flexibility in deceptive message production as compared with non-research deception (Levine, 2018). Therefore, most of the researchers have concluded that the deception paradigms have a greater ecological validity under natural conditions than under controlled conditions (Marvin, 1968; Wright et al., 2012, 2013; Levine, 2018; Kihlstrom, 2021).

Researchers have identified several potential threats to ecological validity. For example, Delgado-Herrera et al. (2021) reviewed the ecological validity of fMRI deception tasks and concluded that “intention to lie” was the component least frequently included in deception tasks, followed by “social interaction,” while “monetary reward” was the most frequent motivation. It may be indicative of the preference for setting monetary incentives for designing deception paradigms. However, tasks with high ecological validity that have used monetary incentives have been shown to recruit fewer brain areas than tasks, with low ecological validity [refer to Delgado-Herrera et al. (2021)]. In addition, the measures with proven discriminability of ecological validity are also difficult to generate (there is no way to test the ecological validity of deception). Although challenging, some related studies have identified several components of deception that have a high degree of ecological validity, such as monetary incentives, motivation, and fear of failure (Wright et al., 2012; Levine, 2018; Delgado-Herrera et al., 2021; Kihlstrom, 2021).

Deceptive behaviors often occur in cross-cultural communications (Levine et al., 2016). In general, findings have revealed that the accuracy of individuals in cross-cultural deception detection is below chance (Taylor et al., 2017). The culture affects a deceiver’s motivations, cognitive difficulty, arousal, and behavioral control. In addition, it also shapes the behavioral patterns relevant to deception (Burgoon et al., 2021). Therefore, cross-cultural deception has also received considerable attention in the deception community (Papantoniou et al., 2021). In particular, Perez-Rosas et al. (2014) revealed that cross-cultural differences are motivated by issues such as languages, beliefs, and moral values, which might influence how the deception is perpetrated and influence the detection of deception. However, most deception research has been undertaken in a “cultural vacuum” of North Americans and Western Europeans, with only a few studies in Asia. According to Levitan et al. (2015), it is problematic that there is a lack of research on deception in cultures other than Western cultures.

In addition, Castillo and Mallard (2012) suggested that low cross-cultural deception judgment accuracy may result from individuals being unfamiliar with the relevant characteristics of the verbal and non-verbal behaviors of people from other cultures. Specific patterns of behavior associated with dishonesty in one cultural context may not be perceived as suspicious behavior in other cultures (Giles et al., 2021). To some extent, detecting lies can be achieved in cross-cultural situations, but a judge must have access to both auditory and visual evidence (Al-Simadi, 2000). While samples based on verbal and non-verbal cues have been examined extensively in English-speaking and Western cultures, few studies have included large numbers of Asian participants (Cheng and Broadhurst, 2005; Levitan et al., 2015).

Researchers have also paid close attention to individual differences in deceptive behavior, including gender (Zhang et al., 2017), age (Ruffman et al., 2012), and personality traits (Semrad et al., 2019). The studies have extensively studied healthy individuals; however, relatively few studies have examined whether lying and truth-telling differ between individuals with brain damage and healthy individuals. Ceceli et al. (2022) reported that drug addiction is associated with prefrontal cortex damage that affects emotional, cognitive, and behavioral functions. The deception and the detection of deception are closely related to these factors (Sporer, 2016). Drug addicts often lie to others to hide signs of addiction, which may lead to severe problems in interpersonal relationships. However, few studies have included a sample of people with drug addiction to research the differences between deception and its detection.

In this study, we develop a novel deception database called C2W2D, a free resource containing 168 videos (84 deceptive videos and 84 honest videos). We review related research and introduce the advantages of the werewolf game approach, which is followed by the development and selection of video clips for inclusion in the database. To the best of our knowledge, the C2W2D filled the gap by developing a Chinese adult deception database under natural conditions (drug addicts and healthy undergraduates), which may enable the exploration of individual and cross-cultural differences in deception research with high ecological validity.

## 2. Related studies

To investigate potential deception cues, a wide variety of deception paradigms have been developed (refer to Table 1). In a related study, we identified two important conditions of deception research: lab-based and natural conditions. We then discussed the advantages of the werewolf game.

**TABLE 1:** Summary of publicly available adult deception database.

	Subjects	Samples	Presentation	Condition	Paradigm	Race
MU3D database (Lloyd et al., 2019)	80	320	Video	Lab	Question	White, Black
Silesian database (Radlak et al., 2015)	101	101	Video	Lab	Question	N/A
DeCour (Fornaciari and Poesio, 2012)	31	2,147	Utterance	Out-lab	Court	N/A
Pérez-Rosas et al. (2015)	56	121	Video	Out-lab	Court	White, Black
Ten Brinke et al. (2014)	6	12	Video	Lab	Mock-crime	N/A
DDD (Huang et al., 2019)	96	27.2 h	Linguistic	Lab	Interactive game	Asian
DSD database (Schuller et al., 2016)	72	162 min	Linguistic	Lab	Mock-crime	N/A
CDC (Levitan et al., 2015)	126	92.3 h	Linguistic	Lab	Interactive game	N/A
Wang et al. (2021)	693	280	Video	Out-lab	Interactive game	Asian, White, Black

### 2.1. Lab-based condition

In previous laboratory studies, participants have been instructed to report accurate or false information regarding their personal facts and opinions. For instance, Evans et al. (2013) asked participants to provide their actual or faked whereabouts at a specified date and time (e.g., “where were you on the last Saturday night from 7:00 to 10:00 p.m.”). Similarly, Radlak et al. (2015) enrolled 101 participants who observed a simple geometric shape with the inscription “truth” or “lie” in sequence on a laptop screen and were asked to either tell the truth or lie about what they saw, yielding 101 videos. Lloyd et al. (2019) recruited 80 participants (black people and white people) to talk about individual events (e.g., “Describe a person you dislike as if you like them”). This database contained 320 videos. In opinion paradigms, participants are instructed to comment on social issues (e.g., “Should convict cold-blooded murderers be executed?”) and then speak truthfully or deceitfully about a particular opinion (Frank and Ekman, 1997; Leal et al., 2010). As controlled methods may generate few lies and unmotivated deceptions, paradigms based on lab conditions may have less ecological validity and are unable to generalize real-life situations (Wright et al., 2013; Delgado-Herrera et al., 2021; Kihlstrom, 2021).

### 2.2. Natural condition

As far as we learned, very few studies have examined deception under natural conditions. While deceptive behaviors are taking place, it is difficult to capture them intentionally. In addition, artificially simulating the deception paradigm with high stakes may engender serious ethical concerns (Fornaciari and Poesio, 2013). Thus, court trials and mock crime paradigms have usually been used to collect datasets. For example, Fornaciari and Poesio (2012) created a corpus including 3,015 utterances (1,202 honest ones, 945 false ones, and 868 uncertain ones) from hearings in Italian courts. Pérez-Rosas et al. (2015) also introduced a database containing 121 video clips (61 deceptive ones and 60 truthful ones) collected from public court trials. In mock crime paradigms, Ten Brinke et al. (2014) asked people (*N* = 12) to tell the truth and lie about stealing $100. Wang et al. (2021) adapted a mafia game for collecting 280 videos, including 110 spies (deception) and 170 villagers (truth).

### 2.3. The advantages of the werewolf game

The C2W2D was developed using the “werewolf” deception game (Hung and Chittaranjan, 2010). The “werewolf” game is a competition between the truth-tellers (villagers and gods) and liars (werewolves). Each player receives an identity (ID) card, which assigns them a role as a god, villager, or werewolf. Please note that this role is not known to anyone except the moderator who directs the game. After the moderator shuffles the cards, he/she hands them face down to each player. Each player looks at their cards in privacy. Four of the 12 players are werewolves who want to slaughter everyone in the village. The other eight players are divided into two groups, such as four gods and four villagers. If any group is killed, the werewolves win the game. On the contrary, if all four werewolves are checked out, the villagers and gods win the game. The game round is divided into “Day” and “Night” alternately, where the round starts at “Night.”

#### 2.3.1. Night phase

The moderator asks all the players to close their eyes and remain quiet. The moderator wakes up the four werewolves. After the werewolves see each other, they secretly discuss which villager or the god they are going to kill. Please note that a consensus must be reached among the werewolves and only one villager or god can be killed. After the finalization of the target, the moderator asks them to close their eyes again. Then, the moderator lets the seer, wizard, hunter, and idiot open and close their eyes one after another and use their skills when they open their eyes (the seer can identify the player and distinguish between a werewolf, villager, and a god; the wizard has one chance to poison the werewolf and one chance to save the villagers, and the hunter has one chance to hunt a werewolf; the idiot has two lives in one game). After the gods have silently decided their actions, all the players enter the “Day” phase of the game.

#### 2.3.2. Day phase

**FIGURE 1:**
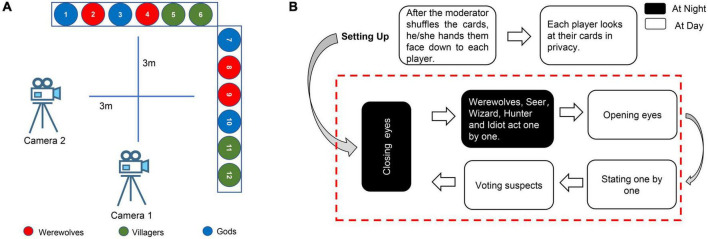
**(A)** The layout of the recording room. Two cameras record the entire game process. The 12 undergraduates or 6–8 addicts sit in front of the camera. Each camera separately captures six undergraduates (three or four addicts). **(B)** The process of one game round. The night and day are looped until one game round had ended.

The moderator instructs all the players to make statements one by one, that is, state who might be a werewolf, who might be a villager, and who might be a god. The players then participate in voting to decide who is most likely to be a werewolf. It is noteworthy that the players can abstain from voting. Once the majority of the players vote for a player to die, the moderator declares that the particular player is dead and the player is shown in their cards. If someone wants to protest his/her innocence or reveal some information, such as the true result of the seer’s vision, he/she must do so before the end of the voting process. No player is allowed to reveal his/her cards to anyone unless the opponent is killed. All the players are not allowed to talk. Once a certain player is voted to death, the night phase is initiated, and the cycle is repeated. This process is continued until one side wins (refer to Figure 1).

Werewolves who cannot tell lies effectively can be exposed as liars very easily and may even be “killed.” On the other hand, if a villager/god cannot convince his/her potential teammates that he or she is telling the truth, they may be thought of being a werewolf and expelled. Even though all the players are capable of lying or telling the truth, few werewolves are able to expose themselves actively to the spotlight. Furthermore, it is important to stay in line with potential teammates. As soon as lying is detected, the logical line constructed by all teammates may be broken, resulting in a loss. In this scenario, the player must pay more cognitive effort and enhance motivation to lie or tell the truth to succeed. Thus, using the “werewolf” game has the following advantages: (1) obtaining samples of deception and honesty without any control and (2) in contrast to traditional one-to-one communication, deceptive and honest behaviors occur in multi-person intergroup communication, which may be closer to reality. In the C2W2D, an additional 10 CNY (Chinese Yuan) is earned if a player presented a fool-proof performance during one round of the game.

## 3. Materials and methods

In the development of C2W2D, two waves are involved, such as data gathering and selection. Two types of samples are categorized, such as lying and telling the truth. The video clips are all publicly available for academic research.

### 3.1. Data gathering

The posters for “playing werewolf game” are displayed in the Student Activity Center of the Jiangxi University of Chinese Medicine. The game fluency and data recording may be limited if the participants do not understand the game rules. Hence, we set up an examination for selecting undergraduate participants (e.g., “Do you know how to play the werewolf game?” and “What can you do if you are a werewolf, wizard, idiot, or villager?”). Upon passing the certification test, one was able to participate in the game. At the Yongqiao Compulsory Isolated Detoxification Center, Jiangxi Province of China, one researcher and three graduates invited drug addicts during the abstinence period to participate in our research. Almost all of the addicts were middle-aged men who could barely play the werewolf game, thus, we practiced with them until everyone could play.

Four groups of drug addicts and five groups of healthy undergraduates participate in the game. During one round of the game, the werewolves are pseudo-randomly^1^ assigned, and the villagers and gods are randomly assigned. Players are not informed of the primary objective of the experiment to avoid introducing subtle emotional changes. We filmed all the players using two high-speed digital cameras (Canon XA20 and JVC CU-VF100AC) at 1,920 × 1,080 at 50 frames per second (refer to Figure 1).

### 3.2. The definition and editing of lying and truth-telling video clip

Raw videos contain irrelevant parts since they are recorded continuously. The type and editing of the target video clips are defined. Three coders from the same lab edit the video clips.

Based on the content of the player’s speech, it is determined which type of video clip is appropriate (lying/truth-telling). In the event that the player’s speech content coincided with his/her identity during the game, the target clip is considered truth-telling. On the other hand, if the player’s speech content did not correspond with his/her assigned identity, it is considered as lying. Each coder knows the real identity of each player (we record the whole game, including the assignment of identity to the player, that is, the experimenter is also the coder, who knows the role played by the participants). Therefore, during the editing stage, the ground truth of each player is known whether they are telling the truth or are lying.

Three coders edit the video clips using Premier software. Each video clip lasts from the beginning to the end of the behavior (action or speech). The video clip is excluded if the screen clarity is compromised, for example, if an irrelevant person passed through the lens of the camera.

## 4. Results

### 4.1. Participants

Participants included 29 Chinese drug addicts (mean age = 36.21, SD = 8.43; 29 men) who were in the abstinence period and 59 healthy Chinese undergraduates (mean age = 20.54, SD = 2.25; 31 women and 28 men).

### 4.2. Video clips information

Raw video footage demonstrating deception is lost for three drug addicts, and raw video footage showing honesty is lost for one undergraduate. To balance the data, the four players are excluded. Thus, the final C2W2D includes data obtained from 26 drug addicts and 58 healthy undergraduates. Approximately 981 video clips are collected (470 deceptive videos and 511 honest videos), including 222 video clips (119 deceptive videos and 103 honest videos) from drug addicts and 759 video clips (351 deceptive videos and 408 honest videos) from healthy undergraduates.

Deception cues may vary from person to person, and signals that indicate lying usually differ from one individual to another (Levine, 2020). Accordingly, by using Premier software, we merged the video clips for each player who told the truth and lied in the honest video clip and a deception video clip. This reduces the variation in individual differences. As a final note, 52 video clips of addicts are stored in C2W2D, including 26 deceptive videos (mean duration = 47.11 s, SD = 38.45 s) and 26 honest videos (mean duration = 40.07 s, SD = 39.07 s), as well as 116 video clips of healthy undergraduates including 58 deceptive videos (mean duration = 250.38 s, SD = 158.54 s) and 58 honest videos (mean duration = 353.52 s, SD = 249.12 s).

### 4.3. The analysis of facial action units

Action units (AUs) refer to the contraction or relaxation of one or more facial muscles [refer to Ekman and Friesen (1978)] that may be used to discriminate between lying and truth-telling.

**FIGURE 2:**
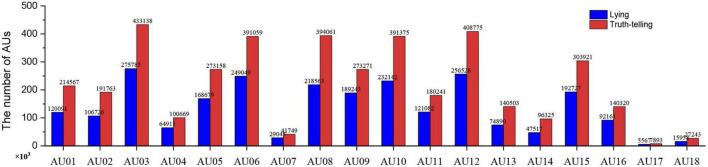
Number of action units (AUs) indicating lying and truth-telling.

**FIGURE 3:**
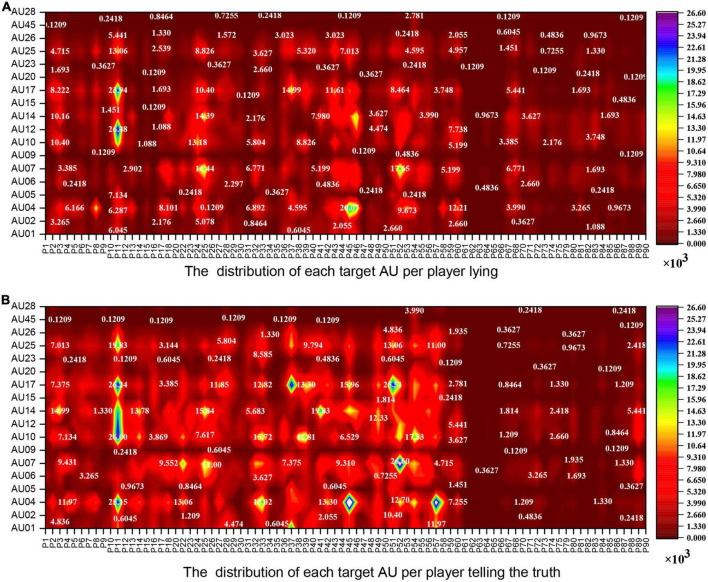
The contour line plot shows the distribution of action units (AUs) per player. **(A)** The distribution of lying AUs per player. **(B)** The distribution of truth-telling AUs per player. P1–P90: The ranking of the players. P1–P60 are healthy undergraduates, P61–P90 are drug addicts. P19, P21, P69, and P76–P78 are precluded in this sequence.

We employ OpenFace2.0 to identify the AUs frame by frame, which is able to identify 18 AUs. Each frame of the video clip is evaluated for the presence of AU (0 or 1) and its intensity (0–5) (for more information, see https://github.com/TadasBaltrusaitis/OpenFace/wiki/Output-Format). There are 1,738,760 frames of facial data output, where 115,229 frames represent drug addicts (62,175 deceptive frames and 53,054 honesty frames) and 1,623,531 frames represent undergraduates (596,939 deceptive frames and 1,026,592 honesty frames). We calculate the total frames of lying and truth-telling within each target AU (refer to Figure 2) using Python. In addition, we employ Python to sum the total frames of lying and truth-telling per player in each target AU (refer to Figure 3).

**FIGURE 4:**
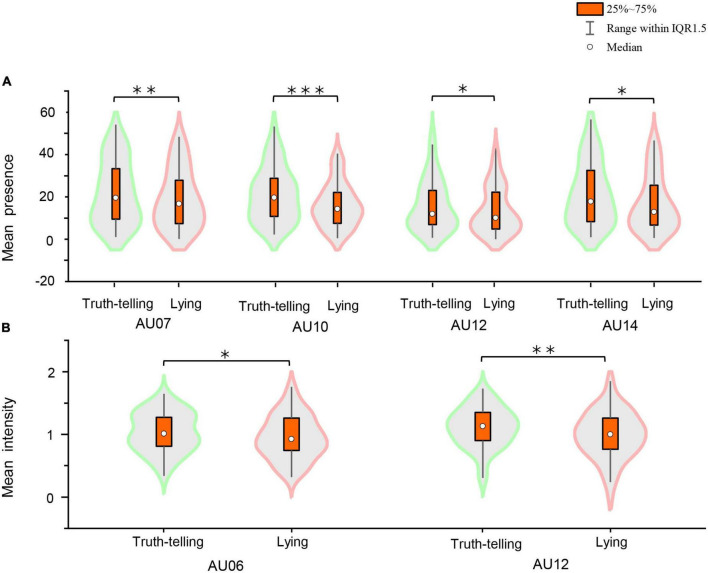
**(A)** Mean presence. **(B)** Mean intensity. Statistically significant differences between lying and truth-telling: *p < 0.05, ^**^p < 0.01, and ^***^p < 0.001.

To explore the lying and truth-telling discrimination of the current database, we calculate the mean presence and mean intensity of each AU per player *via* Python. For the former, the total presence of each AU per player is divided by the corresponding total time of the video clips, and the results of the paired *t*-tests show that there are some significant differences between lying and truth-telling in AU07 (*t* = 2.930, *p* = 0.005^**^, *effect size*^2^ = 0.38), AU10 (*t* = 3.784, *p* < 0.001^***^, *effect size* = 0.50), AU12 (*t* = 2.109, *p* = 0.039*, *effect size* = 0.28), and AU14 (*t* = 3.660, *p* = 0.001^**^, *effect size* = 0.40). For the latter, the total intensity of each target AU per player is divided by the total presence. The results of paired *t*-tests reveal that there are some significant differences between lying and truth-telling in AU12 (*t* = 3.245, *p* = 0.002^**^, *effect size* = 0.43) and AU06 (*t* = 2.419, *p* = 0.019*, *effect size* = 0.32) (see Figure 4). Additionally, in order to avoid multiple-testing problem and Type I error, we set the *p*-value to 0.0025 and employed permutation tests^3^ to examine the AU indicators. The results show that AU10 remains a significant difference (*p* = 0.0008*, *effect size* = 0.45). The same methods are tested on drug addicts and no significant differences are found.

## 5. Discussion

In this study, a new deception database is developed, which effectively addressed the gap of the lack of available samples of deception and honesty of Chinese drug addicts and healthy adults in naturalistic conditions. We developed the C2W2D based on the paradigm of the “werewolf” deception game. In the C2W2D, 1,738,760 frames of facial data are stored in 168 video clips (84 deceptive videos and 84 honest videos) from 26 drug addicts and 58 healthy undergraduates.

An examination of the mean values of presence and intensity of each AU per player is conducted to differentiate lying from truth-telling. As shown in Figure 4, the mean presence of AU07, 10, 12, and 14 and the mean intensity of AU06 and 12 are significantly different in healthy undergraduates. AU06 and 12 are feature vectors of AUs of happiness, and AU07 is one of the components of fear and anger, while AU14 represents the facial muscle of the buccinator (for more details of AUs, please see https://imotions.com/blog/facial-action-coding-system/). However, to control Type I error seriously (we set the *p*-value to 0.0025), the results showed that AU10 was significantly different between deception and honesty. Hence, in the healthy undergraduates participating in this study, the emotion of happiness might be a predictor of deception to some extent. In addition, emotional cues such as fear and anger might also leak from healthy undergraduates in the C2W2D. Moreover, AU10 is a reliable cue for differentiating truth-telling from lying in our database. In contrast, drug addicts did not exhibit significant differences. In our analysis, the numbers of frames of healthy undergraduates are much higher than those of drug addicts (approximately 14:1), which may have led to bias in the drug addicts’ results. Alternately, Goldstein and Volkow (2002) revealed that four circuits in the brain are involved in drug abuse and addiction as follows: (1) nucleus accumbens and ventral pallidum; (2) orbitofrontal cortex and subcallosal cortex; (3) amygdala and hippocampus; and (4) prefrontal cortex and anterior cingulate. Meanwhile, emotional processing appears to be interlocked with perception, cognition, motivation, and action, which is closely related to the amygdala and the prefrontal cortex (Pessoa, 2018). Drug use and dependence, particularly excessive drug seeking and taking, adversely affect these networks with fundamental changes in cognition and emotional processing (Zilverstand et al., 2018; Natale Salvatore et al., 2022). Thus, in the current study, the negative results found in drug addicts may have been caused by leaking different emotional expressions when lying or telling the truth as compared with healthy undergraduates.

Furthermore, studies have extensively investigated emotional expressions of deception in healthy people, while drug addicts who suffer from a range of emotional, cognitive, and behavioral alterations, as well as the desire to escape social problems, often lie (Marvin, 1968; Weir, 1972). However, few studies have tested their leakage of different cues, which is likely due to the lack of a unique database. Thus, we collected drug addicts’ samples for future research.

Although the results of the current study show some significant differences in some AUs, the use of these indicators to detect a liar remains to be tested. Additional potential indicators are also worth mentioning. Shen et al. (2021) found that the total duration of AU20 of fear under the lying condition is less than that under the truth-telling condition, and facial movements around the eyes are more asymmetrical when people are telling lies as compared when telling the truth with high stakes. The pitch of speech and pupil dilation have also been linked to deception in some studies (DePaulo et al., 2003; Levine, 2020). According to Darwin’s proposition, for finding cues to deception, Ekman (2003) proposed the emotional “inhibition hypothesis,” which has gained some empirical traction (Porter and ten Brinke, 2008). Collectively, the leaked emotions can be used as cues of deception, although they are not deception *per se* and are just closely linked with deception.

For common people, detecting deceptive emotional cues is a challenging task. It is, therefore, imperative to use automated deception detection to detect liars quickly and efficiently. A primary method of detecting deception is through the use of computer algorithms, which perform effectively based on big data to build computer vision models. Unfortunately, few deception databases with high ecological validity under natural conditions can be accessed by the public (especially from Asia). Another goal of developing the C2W2D is to enable the extraction of spontaneous micro-expressions, which has attained considerable attention from the deception and emotion research communities. Micro-expressions are reported to be the uncontrolled leakage of an emotional cue, which are estimated to be between 0.04 and 0.5 s in duration (Shen et al., 2012; Yan et al., 2014). While it is difficult to observe micro-expressions with the naked eye, they can be effectively detected *via* computer vision. Furthermore, computer vision algorithms also require an extensive amount of data for micro-expression recognition. However, the existing micro-expression database is developed primarily under laboratory conditions with low ecological validity (Yan et al., 2014; Li et al., 2022). Accordingly, the C2W2D may further evolve into a micro-expression database with a high ecological database. Meanwhile, we consider that catching liars is a process of integrating all the potential deception cues, rather than being limited to a single cue. Thus, while developing the C2W2D, we also intentionally recorded players’ postures and voice cues. As far as we learned, applying multi cues to detect deception currently is a hot topic and can significantly improve the accuracy of detecting deception.

The critical aspect of developing a deception database is how to elicit deception. In other words, what is the most effective method of eliciting deception? As a result of the limitations of the law, data with high ecological validity, such as suspect interrogations, may not be accessible. We, therefore, implement the “werewolf” game deception paradigm under natural conditions. As compared with the traditional methods of developing deception databases, the C2W2D is developed by observing group-to-group interactions in which the identification of telling truths or lies is more complex as compared with one-to-one situations. The competition between the two groups (werewolves vs. gods and villagers) may have resulted in players introducing more authentic emotion leakage and applying more cognitive effort, as well as feeling more fear, which is likely to have contributed to producing deception with high ecological validity.

In the field of deception detection, a critical element for discriminating lies from truths is finding deception cues. Factors, such as ecological validity, cross-cultural differences, and individual differences, can cause the leakage of different emotional deception cues, which have recently received increasing attention within the deception community. It is likely that an important reason for the development of these domains being limited is the lack of adequate samples. Thus, the main aim of this study is to provide a relatively sizable number of deceptive and honest samples from Chinese undergraduates and drug addicts. We do not expect that the C2W2D will replace all the existing deception databases, although it was developed under naturalistic conditions.

## Data availability statement

The raw data supporting the conclusions of this article will be made available by the authors, without undue reservation.

## Ethics statement

The studies involving human participants were reviewed and approved by the Institutional Review Board (IRB) of Jiangxi University of Traditional Chinese Medicine. The patients/participants provided their written informed consent to participate in this study. Written informed consent was obtained from the individual(s) for the publication of any potentially identifiable images or data included in this article.

## Author contributions

CY conducted the experiment, analyzed the data, and wrote the manuscript. XY guided the study. XX, YD, YZ, and BW conducted the data collection. HF, WW, LF, and GH coded the samples and analyzed the data. XS conceived the study and acquired the funding. All authors contributed to the article and approved the submitted version.

## Footnotes

1 Pseudo-random means that if 12 undergraduates play the “werewolf” game, a minimum of three games will be played. After each game ends, we intentionally reset the werewolf identity to ensure that there are no werewolves included in the new game from the previous game (the players are not informed about this). Therefore, by the end of three games, we have 12 people who lied and told the truth. We attempt to make each player that got a chance to play the werewolf (lying). The same method is applied to the drug addicts. The roles of truth-telling (villager and God) are selected randomly throughout the game.

2 We calculated Cohen’s d to measure the effect on size by using G-power software.

3 We used the MATLAB code of permutation test to examine the significant difference of AU (see the following link for details: https://github.com/lrkrol/permutationTest test).
